# Copepod manipulation of oil droplet size distribution

**DOI:** 10.1038/s41598-018-37020-9

**Published:** 2019-01-24

**Authors:** Marco Uttieri, Ai Nihongi, Peter Hinow, Jeffrey Motschman, Houshuo Jiang, Miquel Alcaraz, J. Rudi Strickler

**Affiliations:** 1 Department of Integrative Marine Ecology, Stazione Zoologica Anton Dohrn, Villa Comunale, 80121 Naples, Italy; 2 CoNISMa (National Interuniversity Consortium for Marine Sciences), Piazzale Flaminio 9, 00196 Rome, Italy; 3 School of Freshwater Sciences, University of Wisconsin-Milwaukee, Milwaukee, WI 53204 USA; 4 Department of Mathematical Sciences, University of Wisconsin - Milwaukee, Milwaukee, WI 53201 USA; 5 Department of Mechanical Engineering and Materials Science, Duke University, Durham, NC 27708 USA; 6 Applied Ocean Physics and Engineering Department, Woods Hole Oceanographic Institution, Woods Hole, MA 02543 USA; 7 Institut de Ciències del Mar (CSIC), Passeig Marítim de la Barceloneta 37–49, 08015 Barcelona, Catalonia Spain

## Abstract

Oil spills are one of the most dangerous sources of pollution in aquatic ecosystems. Owing to their pivotal position in the food web, pelagic copepods can provide crucial intermediary transferring oil between trophic levels. In this study we show that the calanoid *Paracartia grani* can actively modify the size-spectrum of oil droplets. Direct manipulation through the movement of the feeding appendages and egestion work in concert, splitting larger droplets (Ø = 16 µm) into smaller ones (Ø = 4–8 µm). The copepod-driven change in droplet size distribution can increase the availability of oil droplets to organisms feeding on smaller particles, sustaining the transfer of petrochemical compounds among different compartments. These results raise the curtain on complex small-scale interactions which can promote the understanding of oil spills fate in aquatic ecosystems.

## Introduction

Recent years have seen increased public awareness about the consequences of oil spills in marine ecosystems. The release of petroleum represents a critical source of environmental risk, with heavy consequences on the life of aquatic organisms^1^. Oil spills can either occur at the sea surface, as in the case of petroleum tankers shipwrecks (e.g., Exxon Valdez; Alaska, March 24, 1989), or underwater, typically owing to deepwater oil well blowouts (e.g., Deepwater Horizon; Gulf of Mexico, April 20, 2010). Recent work in environmental science and marine pollution has focused on both experimental determination and theoretical prediction of the oil droplet size distribution following an underwater well blowout^2^. By turbulent mixing, subsurface spills create oil droplets (ODs): those larger than 5 mm can reach the surface within hours, while rising times for smaller droplets can extend up to 24 h or more^2^. In such a case, OD distribution is additionally influenced by hydrostatic pressure and presence of dissolved gases^3^. In the case of surface spills, the activity of breaking waves breaks up oil slicks into ODs with diameter ranging 10–100 µm^4^.

Chemical dispersants are operationally adopted to facilitate the breaking of surface slicks, limiting the oiling of beaches and coastal areas^5,6^ and enhancing natural degradation by bacteria^7,8^. Dispersants allow the creation of ODs with Ø < 70 μm, which remain suspended in the water for weeks to months owing to colloidal stability^2,5^. It has been estimated that, in the framework of the Deepwater Horizon blowout, 1.84 Mgal of chemical dispersant were applied^9^, and that 16% of spilled oil remained suspended in the form of ODs^10^ upon the application of COREXIT® EC9500A, currently the dispersant of choice^5^.

As a mixture of hydrocarbon and non-hydrocarbon compounds, crude oil and its products can exert strong toxic, carcinogenic, teratogenic and mutagenic effects on aquatic organisms^11–13^. The dimensional and rheological properties of ODs are similar to those of phytoplankton cells, making them eligible “fake prey” for many planktonic organisms^11,14–19^. Pelagic copepods, in particular, are crucial intermediaries between trophic levels, providing the link between primary producers and secondary consumers, but also sustaining the microbial loop and the recycled production through egestion and excretion^20^. Copepods can uptake oil by ingestion as well as by diffusion over the body surface, accumulating petrochemical compounds in egg lipids, egesting them in fecal pellets or even metabolising them^21^. Evidences also demonstrate that ODs can additionally adhere to copepods’ body and limbs^22^. Exposure to oil compounds may lead to long-term bioaccumulation^23^, with acute toxicity effects more intense in temperate species compared to Arctic ones^24^, as well as to the death of the individuals^11,16^. Furthermore, recent studies have demonstrated that long-term bioaccumulation can also result from short-term exposures^23^.

Calculations based on ingestion experiments reported that *Calanus finmarchicus* can accumulate more than 10 mL oil kg^−1^ biomass^25^, whereas model simulations predicted that the same species can reprocess up to 40% of a spilled oil mass, with higher rates in case of subsurface blowouts creating a larger amount of turbulent-mixing driven ODs^22^. Considering that copepods are staple food for other organisms, such as chaetognaths^26^ and fish larvae^27^, the accumulation of ODs within their body may critically contribute to the transfer of oil compounds among trophic levels.

In this study we delved into the role of copepod feeding behaviour in reshaping the size spectrum of dispersed ODs, evidencing the role of potential biological-driven microscale processes in modifying the dimension of droplets in the aftermath of an oil spill. Notwithstanding their reduced body dimensions, pelagic copepods are capable of active movement in the fluid^28,29^; in addition, they have evolved surprising handling techniques to effectively detect and manipulate prey items^30,31^. To address the main goal of the research, an almost monodispersed distribution of ODs (peak Ø = 16 µm) was created using a tailored microfluidic platform. The modifications in the droplet size distribution after 24 h incubation under controlled conditions in presence of 2, 4 and 6 adult female individuals of the calanoid copepod *Paracartia grani* were studied. The results indicate that the mechanical action of copepods (i.e., active manipulation by swimming and feeding limbs and/or egestion) modifies the size spectrum of ODs, shifting it towards smaller Ø. The feasibility of a direct manipulation through feeding limbs is confirmed by numerical calculations evidencing that the energy necessary to split an OD into two daughter droplets can be covered by the actual energy of copepod appendages movement. These novel observations promote our understanding of the biological processes affecting the weathering of spilled oil, and pave the way to further experimentation at microscale level.

## Results

**Figure 1:**
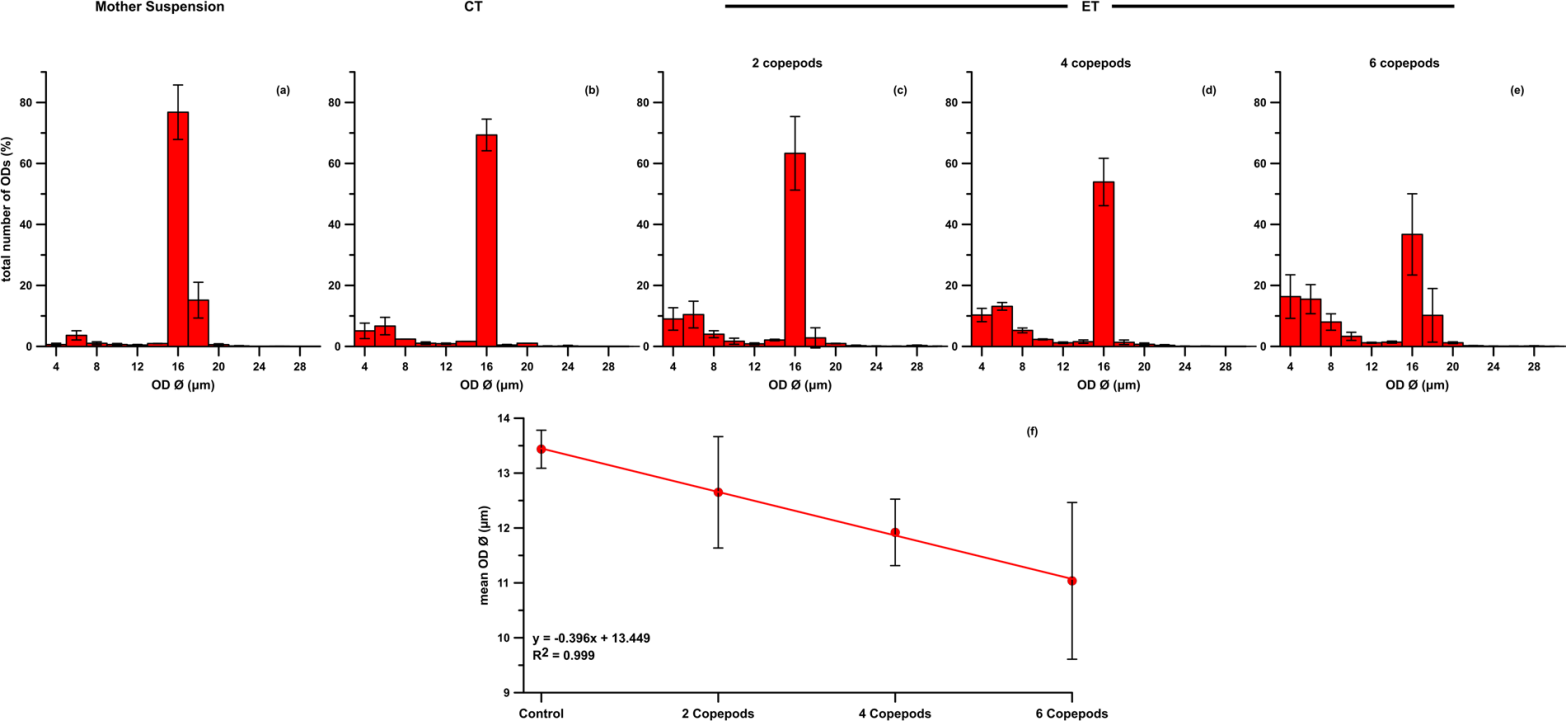
Evolution of OD size distribution. (**a**) Initial distribution in the mother suspension, with marked 16 µm centred monodispersity. (**b**–**e**) ODs size distribution after 24 h in control (**b**; CT) and in experimental treatments (ET) in presence of 2 (**c**), 4 (**d**) and 6 (**e**) *Paracartia grani*. For each treatment, data are pooled among replicates and binned into 2 µm Ø classes. Physical factors determine the reduction of the 16 µm Ø ODs with a concomitant increase in the percent contribution of droplets with Ø = 4–8 µm (CT). The presence of copepods magnifies this process, with an abundance-dependent effect (ETs). Barplots show the mean ± SD values. The relationship between the reduction in mean OD Ø and number of copepods is linear (R^2^ = 0.999) (**f**); for each treatment, the mean ± SD Ø is plotted.

In comparison to the mother suspension, the OD size distribution in CT (see “Methods”) evidenced a reduction in the 16 µm class in favour of an increase in the 4–8 µm range (Figs 1a,b and SI3), with a large (16 µm Ø: *g* = 1.021) to very large (4–8 µm Ø: *g* > 1.300) size effect (Table SI3). The presence of *Paracartia grani* females in ETs (see “Methods”) manifestly emphasised such distributional shift relative to CT (Fig. 1c–e). As the number of incubated copepods increased, a marked reduction in the percentage of 16 µm Ø ODs was scored, in tandem with a concomitant increase in the percentage of smaller ODs ranging 4–8 µm in Ø (Fig. 1c–e). Such an effect was statistically robust as evidenced by both boxplots (Fig. SI3) and Hedges’ *g* (Table SI2). The mean OD Ø decreased linearly as a function of copepod abundance (Fig. 1f; R^2^ = 0.999), although the Kruskal-Wallis *H* test indicated a statistical similarity of the values (*p* = 0.18; confirmed by *post hoc* pairwise analysis). Remarkably, the 10–14 µm diameter range remained scarcely populated.

**Figure 2:**
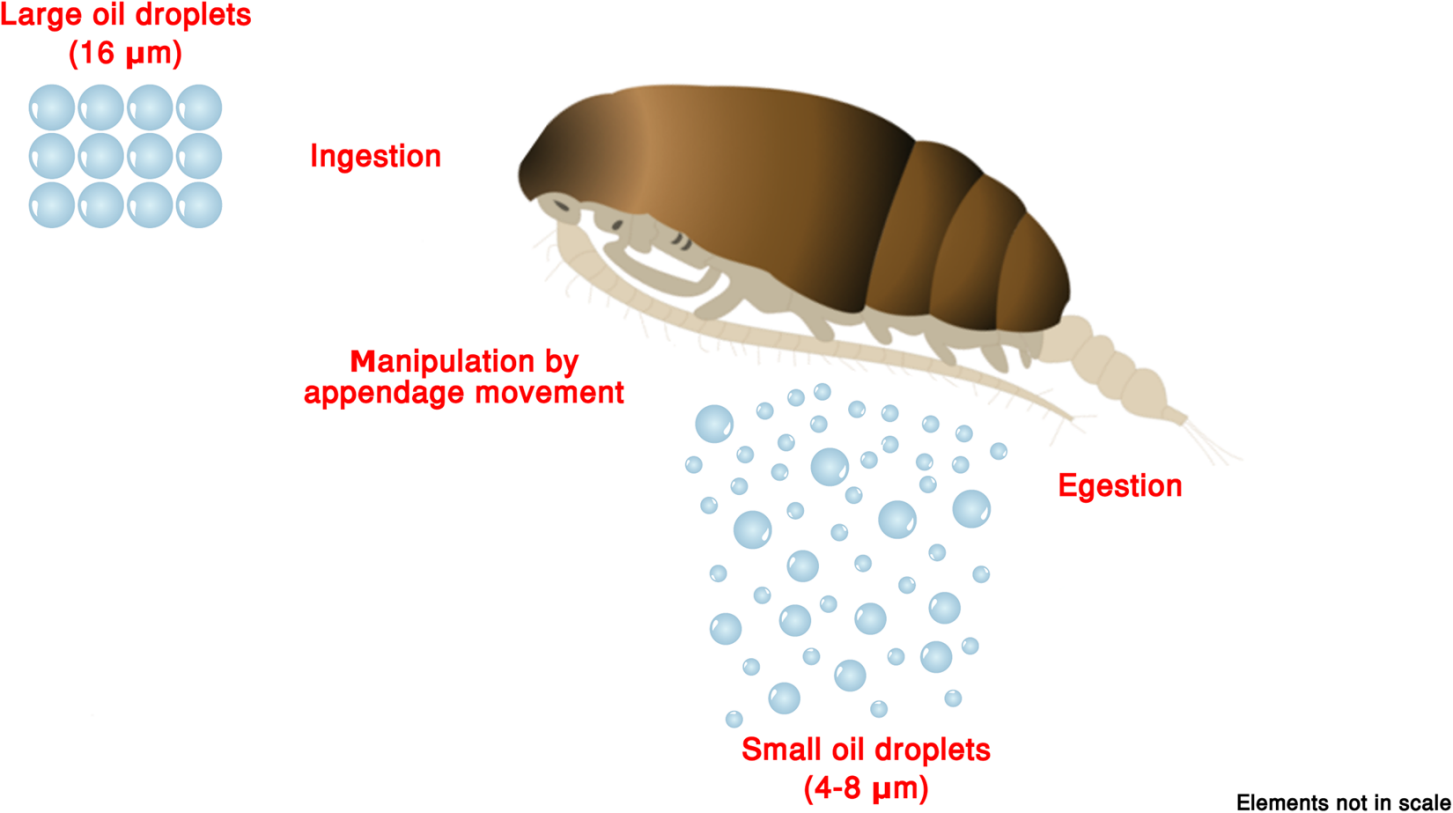
Manipulation of OD size distribution by adult females of *Paracartia grani*: large (16 µm Ø) droplets can be downsized (4–8 µm Ø) by direct manipulation through the swimming and feeding limbs, as well as by egestion processes.

This change in the size spectrum points to the occurrence of a direct manipulation of an OD by two non-mutually excluding processes: 1. the mechanical resizing associated with OD handling by the swimming limbs and the feeding mouthparts, either during ingestion or by active rejection; 2. the egestion of ODs with a reduced diameter compared to that of ingested ones, as discussed in previous works^16^ (Fig. 2).

**Figure 3:**
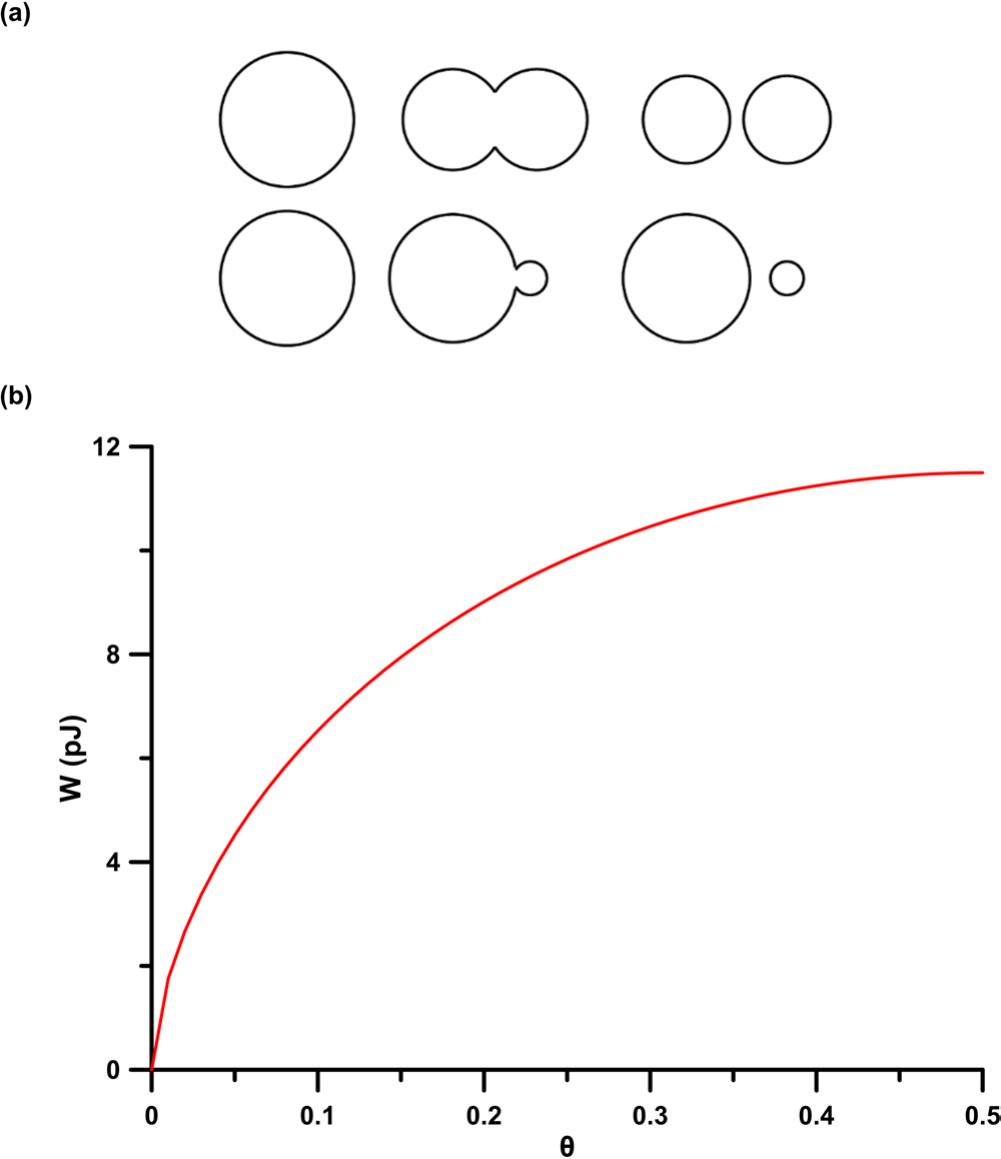
(**a**) Schematic comparison of symmetric (top) and asymmetric (bottom) OD splitting; (**b**) theoretical energy W (pJ) needed to split a 16 µm Ø OD into smaller daughter ODs as a function of the volume fraction of the smaller daughter droplets θ.

If the copepods were able to split an OD with Ø = 16 μm into two daughter ODs of equal volume, these would have Ø ≃ 12.7 μm (Fig. 3a). Looking at the distributions in Fig. 1c–e, it is not conceivable that the so-formed daughter ODs would subsequently be split into smaller and smaller droplets in a symmetric cascade (i.e., any two daughter droplets having the same volume). Instead, we here suggest that the copepods were only able to split small daughter volumes from larger OD (Fig. 3a). Figure 3b shows the calculated interfacial energies to affect a split from a 16 µm Ø OD considering *γ* = 50–55 mN m^−1^ (see Eq. 2 in “Methods” section) as for 5–16 C-atoms n-alkanes^2,32^. Wide ranges of mechanical work values have been reported from different copepod species, in different motion conditions and with diverse bodylengths, such as *Pleuromamma xiphias* (0.1–8 × 10^−7^ J)^33^ and *Cyclops scutifer* (120 × 10^−12^ J)^34^. Using CFD simulations for *Diaptomus minutus*, a suspension-feeding species with a body length comparable to *P*. *grani*, the mechanical power cost can be estimated in the range of 2–6 × 10^−9^ W^35^. Assuming a copepod-OD interaction time <1 s^31^, the energy output of the feeding current would be on the order of 10^−9^* J* (O *pJ*). The curve shown in Fig. 3 indicates that even the smallest amounts of work would make it possible for a copepod to split a 16 µm Ø OD into equal daughter droplets. However, the interfacial energy from Eq. 2 (see “Methods“ section) is only a lower bound, since the copepod needs to spend energy to generate a feeding current transporting ODs to its capture area. Moreover, it may be rare that the copepod hits the oil droplet right in the middle or that upon such a hit the droplet escapes, rather than being broken apart.

The calculation of OD volumes (V_ODs_, µL) demonstrated that the oil volume was conserved among treatments (CT: 1.07 ± 0.09 µL; ET – 2 individuals: 1.09 ± 0.16 µL; 4 individuals: 0.94 ± 0.11 µL; 6 individuals: 0.90 ± 0.05 µL). The Kruskal-Wallis *H* test returned *p* = 0.22, indicating no difference in V_OD_ between CT and ETs. This result was backed up by the *post hoc* pairwise analysis. The conservation of V_OD_ suggests that only a negligible part of the oil was retained by copepods’ guts, and supports the effectiveness of the manipulation process leading to a change in OD size distribution. It is worth noting that, upon visual inspection after the completion of the experiments, no trace of ODs was evident on vials walls.

## Discussion

Oil spills are presently one of the major sources of pollution in aquatic ecosystems^1^, requiring the adoption of integrated environmental management and recovery procedures^36^. The application of dispersants constitutes a response option, favouring the breakup of oil slicks into droplets which are more easily colonisable by oil-degrading bacteria^6,37^. Small droplets, however, can interact with a large variety of zooplanktonic organisms^14,15,17,18,38^, with potential repercussions on other compartments of the aquatic ecosystem.

The results of the present experimentation confirm that ODs can be resized owing to small-scale physical factors associated with the movement of the fluid environment, replicating mixing processes acting in the ocean (CT). Physically-driven size shift can be accompanied by biologically-driven processes. In this framework, this study demonstrates that *Paracartia grani* manipulation can affect OD size distribution, breaking 16 µm Ø droplets into smaller ones. Two plausible, concurrent mechanisms can be identified:

- an active splitting of ODs by *P*. *grani* swimming/feeding appendages, with the creation of smaller droplets;
- the ingestion of ODs, mistaken for prey items, and their subsequent egestion as smaller droplets not included in fecal pellets.

The OD size distributions observed in ETs clearly show that, as the number of incubated copepods increases, the OD dimensional spectrum is progressively shifted towards smaller Ø, in particular in the 4–8 µm window. The calculated energy required to actively cut an OD could be realistically covered by *P*. *grani* appendage movement. In particular, the biased shift observed can be justified considering that the energy required to detach a small parcel from a bigger OD is lower than that required to split a droplet in two symmetric halves. Furthermore, considering *P*. *grani* clearance rate at high food concentration^39^, the recursive manipulation of increasingly smaller ODs supports the observed shift in OD size distribution. The resizing operated by *P*. *grani* closely recalls the disaggregation of marine snow aggregates operated by *Euphasia superba* through small-scale shear generated by its swimming activity^40^. Such commonality supports the ability of zooplankters to actively modify the size spectrum of material suspended in the water column.

Swallowed ODs can instead be resized in the gut, being encapsulated in fecal pellets and/or defecated as such^16^. In this second case, their Ø would likely be <10 µm^16^, in line with the dimensional range scored in the present investigation. The egestion of ODs as such might have been emphasized by the absence of food during the incubation period. Notwithstanding this, the increase in ODs with Ø = 4–8 µm scored in the present investigation provides an experimental support to previous observations^16^, and validate the feasibility of such a resizing process operated by copepods. It is worth underlining that the 24 h incubation period used in the experiments is fully compatible with the gut passage and evacuation time of congeneric *Acartia* species, ranging from 11 to 97 min (*Acartia clausi*^41^; *Acartia tonsa*^42^; *Acartia hudsonica*^43^).

The creation of a monodispersed suspension of ODs is fundamental to investigate the interaction of copepods with prey-like droplets. The microfluidic device implemented in the present work efficiently built a well-peaked distribution of ODs, equalling the efficiency of a similar experimental device^44^. This setup represents a crucial step forward compared to other OD forming systems such as magnetic stirring, which creates an oil emulsion without an accurate definition of droplet size^11,14,16^.

Over the last years, mounting evidence has gathered about the role of copepods as stepping stones in the transfer of plastic debris across the food web^45,46^. The present investigation provides new evidence on the interactions between copepods and ODs, which may affect the availability of petrochemical compounds to organisms feeding on smaller particles. Copepod manipulation produces smaller droplets falling in a range accessible to protozoans^17^, copepod nauplii^14,15^, meroplankton larvae^38^ and pelagic tunicates^18^. As many of these organisms may be preyed upon by copepods, OD manipulation can determine a negative feedback on copepods themselves, as they may re-ingest oil they had already manipulated. Furthermore, oil dispersion may affect copepod vertical distribution, as demonstrated for *Calanus finmarchicus* copepodites^47^. Smaller ODs can additionally be colonized by hydrocarbon-degrading bacteria^7,8^; in particular, droplets with Ø ≃ 5 µm are degraded at a faster pace compared to larger ones (Ø = 100 µm)^48^. The encapsulation of ODs inside copepod fecal pellets sustains the transfer of oil to microbes and benthic organisms^16^, while bacteria indigenous to copepod guts are responsible for the degradation of oil entrapped in fecal pellets^49^.

The exposure to sublethal concentrations of crude oil can reduce the reproduction and egestion rates in different copepod species^14^, and can also result in significant higher mortality^11,14,15^. Toxic effects are further aggravated by the presence of UVB radiation^11,15^, as well as by the presence of oil dispersants^15^. The adoption of a non-toxic paraffin oil excluded lethal effects or behavioural/physiological impairments on tested individuals. Such experimental procedure allowed testing the potential ability of copepods to manipulate ODs. The overall impact in the field may however be less pronounced, as copepod activity might be lowered owing to toxic effects of crude oils. The manipulation ability of copepods may additionally be dependent on the specific physical properties (e.g., density, viscosity, surface tension) of the spilled oil.

Based on modelling and laboratory studies, the mean Ø of droplets created upon massive release of oil after spills or shipwrecks spans several order of magnitude, from μm to mm^50–53^. The unanticipated results of the present research elucidate key mechanisms by which copepods can contribute to the creation of ODs in the lower end of the dimensional spectrum. This process sheds new light on the physical-biological interactions occurring at the microscale, and fosters research on the direct and indirect interactions between ODs and planktonic organisms.

## Methods

Adult females of *Paracartia grani*, a calanoid copepod frequent in neritic marine environments, were used for the experiments. These suspension-feeding copepods are routinely maintained under culture by the Zooplankton Ecology Group at the Institute of Marine Sciences of Barcelona (CSIC), where the experiments were performed. The species was chosen due to the existing broad information about its ecological role in marine ecosystems, from the interaction between zooplankton and physical variables, to its feeding role and mechanisms and the effects of food on growth, production, survival rates, and even ageing^54^. Individuals were cultured in the laboratory at 19.0 ± 0.1 °C and fed on *Rhodomonas salina*.

A custom microfluidic platform generated size-controlled droplet distributions. ODs were created using Hodernal® (Mylan Pharmaceuticals SL, Barcelona, Spain), an inert, non-toxic paraffin oil, and 2% Tween 80 (Sigma-Aldrich, St. Louis, MO, USA), a non-ionic saccharide-based surfactant included in COREXIT® EC9500A^5^. Crude oils have demonstrably lethal effects on copepods when massively present in the environmental fluid, but even at sublethal concentrations they may exert negative impacts, for example lowering feeding rates^55^, reducing egg production, egg hatching and egestion rates^14^, and impairing mating success^56^. For the experiments discussed in this work, the use of the non-toxic Hodernal® allowed focusing on the capacity of copepods to modify OD size distribution without introducing potential elements of variability due to the intrinsic toxicity of crude oils.

Crude oils have a wide range of densities and viscosities, depending on several factors including geographic origin and chemical composition. Based on manufacturer’s data sheet, considering a relative density ρ_oil_ = 0.850 g mL^−1^ at 20 °C the API gravity (°) for Hodernal® can be calculated as^57^:

1 $$API\,gravity=\frac{141.5}{SG}-131.5$$

being $SG=\frac{{\rho }_{oil}}{{\rho }_{water}}$ the specific gravity of the oil compared to that of water (ρ_water_). Since Eq. 1 is calculated at a reference temperature of ~15.5 °C (60 °F), assuming negligible changes in ρ_oil_ between 20 °C and 15 °C (O 10^−3^) and considering ρ_water_ = 0.999 g mL^−1^, Hodernal® API gravity equalled 34.8° (SG = 0.851), a value typical of light crude oils^58^. As a reference, for Macondo crude oil spilled from Deepwater Horizon rig typical values score ρ_Macondo_ = 0.820–0.860 g mL^−1^ resulting in API gravity ranging 32.8°–40.8°^59–61^. Based on these values, Hodernal® could be used as non-toxic substitute for light crude oils^58^. At the end of the incubation period, the vitality and health of *P*. *grani* were visually checked to verify the absence of stress-induced behaviour.

A mother suspension of ODs (mean Ø: 16 µm; Fig. 1a) was generated and diluted in 0.2 µm filtered seawater to an equivalent concentration of ~13,000 ODs mL^−1^, corresponding (as ppm) to that of the saturating ingestion for *P*. *grani* feeding on *R*. *salina*. The OD concentration fell within the range of Ø tested for ingestion experiments in doliolids^18^. The selected Ø (16 µm) was also representative of the typical major axis (length) of the ovoidal *R*. *salina* (10–14 µm)^62^, allowing the creation of ODs mimicking a typical *P*. *grani* prey item. More technical details on the setup are provided as Supplementary Information.

All incubation experiments were performed at the same OD concentration using filtered seawater without food. 30 mL OD-containing vials were filled with 2, 4, or 6 copepods per triplicate (experimental treatments, ETs). Two vials without copepods were used as control (CT). The experimental volumes were equivalent to those tested in previous *P*. *grani* grazing^63^ and ageing^54^ experiments. All vials were attached to a plankton wheel rotating at 0.2 rpm, and incubated 24 h in a temperature-controlled chamber (19.0 ± 0.1 °C) at 12:12 light:dark photoperiod. To view and count droplets before and after incubation, three aliquots (1 mL) of the suspensions were pipetted from each vial into a glass Sedgewick counting chamber. 50 random pictures were taken for each aliquot, equalling 150 pictures for each vial (450 pictures for each treatment), and ODs were automatically counted and measured using ImageJ^64^. As the resolution of the CCD microscopy camera used in the experiments approached ~1–2 µm, and considering the necessity of using specific dyes to identify microscopic artefacts (e.g., debris, dust), ODs with Ø < 4 µm could not be safely quantified. As a consequence, 4 µm was used as lower threshold for ODs analysis.

OD counts from the three aliquots of each single vial were grouped together and binned into 2 µm wide Ø classes, which were subsequently pooled among replicates. Boxplots^65,66^ were used to compare the percent contribution of the most predominant OD Ø classes among the different experimental conditions tested. This was complemented by the measurement of the effect size^67^ by which quantifying the difference between the groups. In particular, owing to the small sample size, the measure of the effect size was calculated using Hedges’ *g*^68^. Depending on *g* values, differences between means were ranked from small to very large, following the criteria established in the literature^67,69^. More details are provided in the Supplementary Information.

When a spherical oil droplet of volume $V=\frac{4\pi \,{r}^{3}}{3}$ is split into two spherical droplets of volumes $\theta \,V$ and $(1-\theta )\,V$, $0\le \theta \le 1/2$ representing the volume fraction of daughter droplets, the total area of the daughter droplets is given by ${\rm{\Delta }}A=4\pi \,({\theta }^{2/3}+{(1-\theta )}^{2/3}-1)\,{r}^{2}$, To investigate the ability of copepods to split ODs through the movement of swimming appendages, the amount of energy *W* (*pJ*) needed to increase the surface area $\,{\rm{\Delta }}{\rm{A}}$ from one mother to two daughter droplets was calculated as^70^:

2 $$W=\gamma \,{\rm{\Delta }}A$$

where *γ* represents the surface tension (mN m^−1^) and ${\rm{\Delta }}{\rm{A}}$ is a function of the volume fraction of smaller daughter droplet (θ). Typical *γ* values for n-alkanes with 5–16 C-atoms range between 50 and 55 mN m^−1^^32^.

As a proxy for mass balance, for each 1 mL aliquot the total OD volume (V_OD_) was calculated starting from the associated OD size distribution and pooled among treatments. Owing to the small sample size (k = 4, N = 11; k: number of samples, N: total sample size), normality tests could not be reliably used to reject the null hypothesis. In such conditions, a nonparametric ranking test should thus be used^71^. Basing on the threshold values for k and N^72,73^, the statistical comparison of the V_OD_s calculated for CT and ET treatments was carried out using a Kruskal-Wallis *H* test^74^ returning a *p* value for the rejection of the null hypothesis that none of the k groups statistically dominated over the others. However, since the Kruskal-Wallis is an *omnibus* test, a *post hoc* test was mandatory to control the inflation of type I errors. Since samples were independent and with unknown distribution, a multiple pairwise comparison procedure using Dunn-Šidák’s correction^75,76^ was employed. The same statistical approach was used to compare the mean OD Ø among the different treatments.

## Supplementary information

Supplementary Information

Video S1

Video S2

Video S3
