# Supplementary material for: Copepod manipulation of oil droplet size distribution
Source: Sci Rep. 2019 Jan 24;9:547. doi: 10.1038/s41598-018-37020-9 (PMC6346107; doi:10.1038/s41598-018-37020-9)
Supplement: Supplementary file 1 — Supplementary Information [file 41598_2018_37020_MOESM1_ESM.pdf]

Supporting Information

"Copepod manipulation of oil droplet size distribution"

Marco Uttieri[1,2], Ai Nihongi[3], Peter Hinow[4], Jeffrey Motschman[5], Houshuo Jiang[6], Miquel Alcaraz[7], J. Rudi Strickler[3]

[1] Department of Integrative Marine Ecology, Stazione Zoologica Anton Dohrn, Villa Comunale, 80121 Naples, Italy

[2] CoNISMa (Consorzio Nazionale Interuniversitario per le Scienze del Mare), Piazzale Flaminio 9, 00196 Rome, Italy

[3] School of Freshwater Sciences, University of Wisconsin-Milwaukee, Milwaukee, WI 53204, USA

[4] Department of Mathematical Sciences, University of Wisconsin - Milwaukee, Milwaukee, WI 53201, USA

[5] Department of Mechanical Engineering and Materials Science, Duke University, Durham, NC 27708, USA

[6] Applied Ocean Physics and Engineering Department, Woods Hole Oceanographic Institution, Woods Hole, MA 02543, USA

[7] Institut de Ciències del Mar (CSIC), Passeig Marítim de la Barceloneta 37-49, 08015 Barcelona, Catalonia, Spain

*** Corresponding author:** Marco Uttieri (marco.uttieri@szn.it), phone: +39 081 583 3212, fax: +39 081 764 1355

Microfluidic device

Microfluidics is an established and continually growing field dealing with the behaviour of fluids at the microscopic level. In droplet-based microfluidic technology, cells are separated and encapsulated into aqueous droplets and are carried in an oil phase (water-in-oil emulsion). This technique is advantageous since these droplets then act as individual micro bio-and-chemical reactors. Additionally, droplet-based techniques using oil-in-water emulsions are spreading as well. Droplet-based microfluidics enable the precise control of particle size: droplets sizes are finely tuned within a fluidic channel by adjusting relative flow rates and microfluidic orifice size [1].

The use of oil-in-water devices to produce droplets with Ø<50 µm is particularly challenging for a host of reasons: 1. these microfluidic systems are particularly prone to clogging; 2. the compatibility of the microfluidic chip material (e.g., PDMS) is low with certain oils (e.g., crude oil); 3. PDMS devices have limitations in terms of operating pressures and suffer leaking. To tackle these issues, a microfluidic platform consisting of glass chips, pressure-driven pumps and experimental fluids was specifically developed to generate size-controlled droplet distributions. Upon preliminary laboratory tests (not shown), glass chips resulted more reliable, robust and chemically resistant than PDMS ones. In addition, pressure-driven pumps were more effective than syringe-based injection systems, providing pulseless flow. Custom devices fabricated by Dolomite Microfluidics (Royston, UK), using photolithography and a wet etching hydrofluoric acid (HF) in a cleanroom facility, were used. The droplet microfluidic chips tested were: small droplet chip (14 µm etch depth), droplet junction chip (100 µm etch depth), and droplet junction chip (190 µm etch depth) (Table SI1 and Figure SI1). Due to the hydrophilic nature of glass and the adoption of the oil-in-water approach, no coating on the chips was required. A Dolomite Microfluidics demonstration video showing the process of droplet creation is available at the URL: https://www.youtube.com/watch?v=GsZovqbujwQ.

The creation of droplets was possible through the use of two phases. The droplet phase consisted of the oil substance (Hodernal®; Mylan Pharmaceuticals SL, Barcelona, Spain), while the carrier fluid phase was made by 2% Tween 80 (Sigma-Aldrich, St. Louis, MO, USA) in seawater. Droplets size were determined primarily by the etch depth and cross-sectional area of each chip, together with flow rates, pressure, and off-chip fluidic resistance. Since the aim was to reconstruct droplets with a diameter of 16 µm (i.e., comparable to that of phytoplankton prey), a small droplet chip (14 µm etch depth) was used which, by accurate tuning of the components, returned well peaked monodispersity (Figure 1 in the main article; Video SI1). Satellite droplets, or secondary emulsions caused by the breaking of the droplet fluid jet due to the Raleigh-Plateau instability [2], are traditionally present in droplet-based microfluidic systems. Satellites droplets are smaller than the parent emulsion and overall lower the monodispersity of droplet populations. Due to the required monodispersity restriction of the experimental protocol, satellites were minimized by avoiding multiple droplet pinch-off points.

By further experimentation, the device demonstrated its ability to create additional droplet distributions within different diameter ranges (small droplets: 5-35 µm Ø; large droplets: 70-150 µm Ø and 150-300 µm Ø) by appropriate tuning of the components of the microfluidic system (Table SI1 and Figure SI1).

Experimental design

A synthetic diagram depicting the experimental procedure is shown in Figure SI2. The microfluidic device provided the monodispersed mother suspension of ODs with Ø=16 µm, at a concentration of ~13,000 ODs mL^{-1}. Such suspension was used to fill the experimental vials (30 mL), which were incubated 24 h on a rotating plankton wheel (0.2 rpm) at 19.0±1.0°C with a 12:12

light:dark photoperiod. For each treatment, 450 random pictures were taken (150 pictures for each vial), for an approximate total of 7,000 ODs measured under the microscope and profiled with ImageJ.

The ability of *Paracartia grani* to ingest 16 µm Ø OD was preliminarily verified by creating droplets enriched with DMS and adding one drop of Nile red (Sigma N-3013, 2 µg /ml) in acetone to 100 mL of the OD mother suspension. Nile red stained droplets fluoresced yellow under the epifluorescence microscope (excitation λ: 500 nm) and could be easily identified and distinguished from other microdroplets. Microscope observations highlighted the ability of *P. grani* to feed on oil droplets, which were clearly recognisable in their gut (Video SI2 and SI3).

Statistical analysis of OD size distribution

The analysis of the size spectrum of ODs from the mother suspension, CT and ETs highlighted two major dimensional components: the 16 µm class, corresponding to the peak Ø of the monodisperse mother suspension; ant the 4-8 µm class, whose percentage contribution to the OD distribution depended upon the different experimental conditions. The results were statistically compared using boxplots [3,4], where each box graphically corresponds to one sample, with the line inside it representing the median value and the bottom and top being the 25th and 75th percentiles, respectively. The boxplots for the 16 µm and 4-8 µm classes are shown in Figure SI3.

To further appreciate to which extent two samples might differ, Hedges' *g* was calculated as [5]:

$$g = \frac{m_c - m_t}{sd_{pooled}} \qquad \text{(SI1)}$$

m_c and m_t being the mean values of the control and the treatment, and sd_{pooled} the pooled standard deviation calculated as [5]: $sd_{pooled} = \sqrt{\frac{(n_c - 1)\, sd_c^{\,2} + (n_t - 1)\, sd_t^{\,2}}{n_c + n_t - 2}}$, where n_c and n_t are the sample sizes for control and treatment while sd_c and sd_t their respective standard deviations. The ranking of *g*-based differences was based on the thresholds set in the literature [6,7].

References

1 Anna, S. L., Bontoux, N. & Stone, H. A. Formation of dispersions using "flow focusing" in microchannels. *Appl. Phy. Lett.* **82**, 364-366, doi:10.1063/1.1537519 (2003).

2 Henderson, D. M., Pritchard, W. G. & Smolka, L. B. On the pinch-off of a pendant drop of viscous fluid. *Phy. Fluids* **9**, 3188-3200, doi:10.1063/1.869435 (1997).

3 Tukey, J. W. *Exploratory Data Analysis*. (Addison-Wesley, 1977).

4 McGill, R., Tukey, J. W. & Larsen, W. A. Variations of box plots. *Am. Stat.* **32**, 12–16 (1978).

5 Hedges, L. V. & Olkin, I. *Statistical Methods for Meta-Analysuis*. (Academic Press, Inc., 1985).

6 Cohen, J. *Statistical Power Analysis for the Behavioral Sciences*. (Lawrence Erlbaum Associates, 1988).

7 Rosenthal, J. A. Qualitative descriptors of strength of association and effect size. *J. Soc. Serv. Res.* **21**, 37-59, doi:10.1300/J079v21n04_02 (1996).

Table SI1: technical details of the microfluidic devices implemented for the creation of small (5-35 µm Ø) and large (70-150 µm Ø; 150-300 µm Ø) oil droplets. Data presented upon permission by Blacktrace Inc. (MA, USA).

Small Droplets Setup	
Materials	
Pump (Pressure-Driven)	2× Mitos P-Pump (PN: 3200016)
Tubing	FEP Tubing 1/16” OD×0.1 mm ID, 10 meters (PN: 3200300)<br>FEP Tubing 1/16” OD×0.25 mm ID, 10 meters (PN: 320063)
Shut-Off Valves	3× 2-way inline valve (PN: 3200087)
T-Connector, ETFE	1x T-Connector ETFE (PN: 3000397)
Microfluidic Chip	Small Droplet Chip (14 µm etch depth) (PN: 3200136)
Droplet Diameter Range	5–35 µm
Connection Type	Top
Chip Holder Interface	1× Top Interface 4-way (4 mm) (PN: 3000109)
Tubing Holder Interface	1× Linear Connector 4-way (PN: 3000024)
Max. Droplet Frequency	~ 10,000 droplets/sec
Tubing Connection Setup	
Aqueous Phase	
Pump ⟶ T-Connector	1× 0.1×~500 mm (inner diameter × length)
T-Connector → Chip	2× 0.25×~ 500 mm (inner diameter × length)<br>(shut-off valve placed half-way between)
Organic Phase	
Pump → Chip	1× 0.25×~500 mm (inner diameter × length)<br>(shut-off valve placed half-way between)
Exit	
Chip → Collection Container	1× 0.25×~300 mm (inner diameter × length)
Supplementary Info	
Chip Material	Glass
Channel Coating	None (hydrophilic)
Chip Size (length × width × thickness)	22.5×15×4 mm
Channel cross-section at junction (depth × width)	14×17 µm
Wide channel cross-section (depth × width)	14×500 µm

Large Droplets Setup	
**Materials**	
Pump (Pressure-Driven)	2× Mitos P-Pump (PN: 3200016)
Tubing	FEP Tubing 1/16” OD×0.1 mm ID, 10 meters (PN: 3200300)<br>FEP Tubing 1/16” OD×0.25 mm ID, 10 meters (PN: 320063)
Shut-Off Valves	3× 2-way inline valve (PN: 3200087)
T-Connector, ETFE	1× T-Connector ETFE (PN: 3000397)
Plug	Plug 1.6 mm (pack of 10) (PN: 3000056)
Microfluidic Chip	Droplet Junction Chip (100 µm etch depth) (PN: 3000158)<br>Droplet Junction Chip (190 µm etch depth) (PN: 3000436)
Droplet Diameter Range	70–150 µm (100 µm etch depth)<br>150–300 µm (190 µm etch depth)
Connection Type	Edge
Chip Holder Interface	1× Chip Interface H (PN: 3000155)
Tubing Holder Interface	2× Linear Connector 4-way (PN: 3000024)
Max. Droplet Frequency	~ 1,000 droplets/sec (100 µm etch depth)<br>~ 500 droplets/sec (190 µm etch depth)
**Tubing Connection Setup**	
**Aqueous Phase**	
Pump ⟶ T-Connector	1× 0.1×~500 mm (inner diameter × length)
T-Connector → Chip	2× 0.25×~ 500 mm (inner diameter × length)<br>(shut-off valve placed half-way between)
**Organic Phase**	
Pump → Chip	1× 0.25×~500 mm (inner diameter × length)<br>(shut-off valve placed half-way between)
**Exit**	
Chip → Collection Container	1× 0.25×~400 mm (inner diameter × length)
**Supplementary Info**	
Chip Material	Glass
Channel Coating	None (hydrophilic)
Chip Size (length × width × thickness)	22.5×15×4 mm
Channel cross-section at junction (depth × width)	100x105 µm (100 µm etch depth)<br>190x195 µm (190 µm etch depth)
Wide channel cross-section (depth × width)	100x300 µm (100 µm etch depth)<br>190x390 µm (190 µm etch depth)

Table SI2: Hedges'*g* measures of the effect size between the different experimental conditions tested. Terminology follows the thresholds discussed in the literature [6,7].

Ø=16 µm	Mother Suspension	CT	ET 2 copepods	ET 4 copepods
**Mother Suspension**	-			
**CT**	1.021***	-		
**ET – 2 copepods**		0.649**	-	
**ET – 4 copepods**		>1.300****	0.906***	-
**ET – 6 copepods**		>1.300****	>1.300****	1.023***

Ø=4-8 µm	Mother Suspension	CT	ET 2 copepods	ET 4 copepods
**Mother Suspension**	-			
**CT**	>1.300****	-		
**ET – 2 copepods**		1.230***	-	
**ET – 4 copepods**		>1.300****	0.742**	-
**ET – 6 copepods**		>1.300****	0.863***	0.617**

Thresholds [6,7]:

*: small **: medium ***: large ****: very large

Figure Captions

Figure SI1: diagrams (a) and pictures of the chip junctions (b) of the microfluidic devices used for the creation of small (5-35 µm Ø) and large (70-150 µm Ø; 150-300 µm Ø) oil droplets. Diagrams reproduce upon permission by Blacktrace Inc. (MA, USA).

Figure SI2: schematic of the experimental design used for incubation experiments.

Figure SI3: boxplots of the percent contribution to the total OD population for the most dominant dimensional classes (a: Ø=16 µm; b: Ø=4-8 µm) in the different experimental conditions tested. The absence of overlap of the medians (lines inside the boxes) and the minimal comparability of the dispersion of data (interquartile range) indicate that the samples are statistically independent.

Figure SI1

(a)

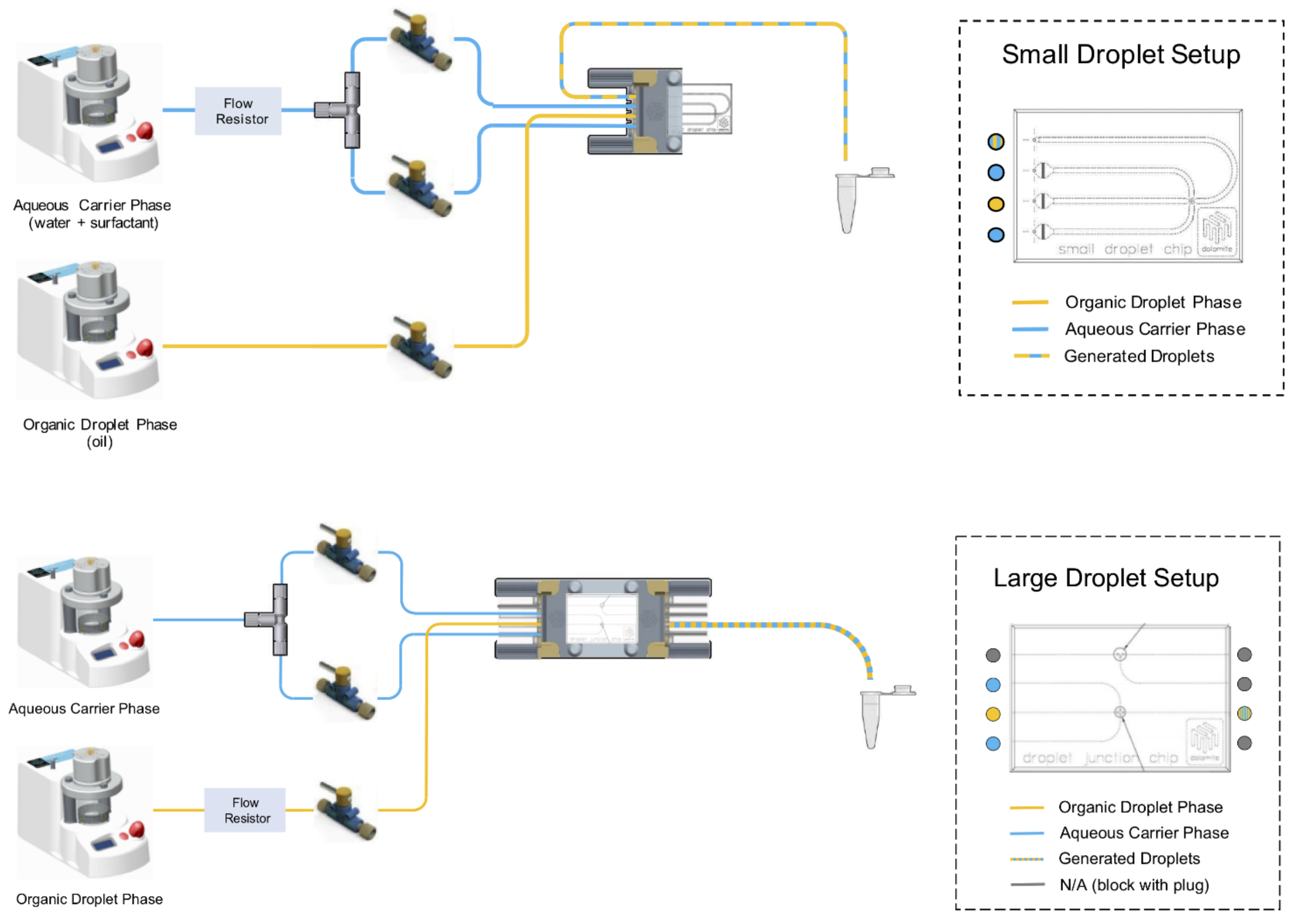
Flow Resistor
Aqueous Carrier Phase (water + surfactant)
Organic Droplet Phase (oil)
Small Droplet Setup
small droplet chip
Organic Droplet Phase
Aqueous Carrier Phase
Generated Droplets
Aqueous Carrier Phase
Flow Resistor
Organic Droplet Phase
Large Droplet Setup
droplet junction chip
Organic Droplet Phase
Aqueous Carrier Phase
Generated Droplets
N/A (block with plug)

(b)

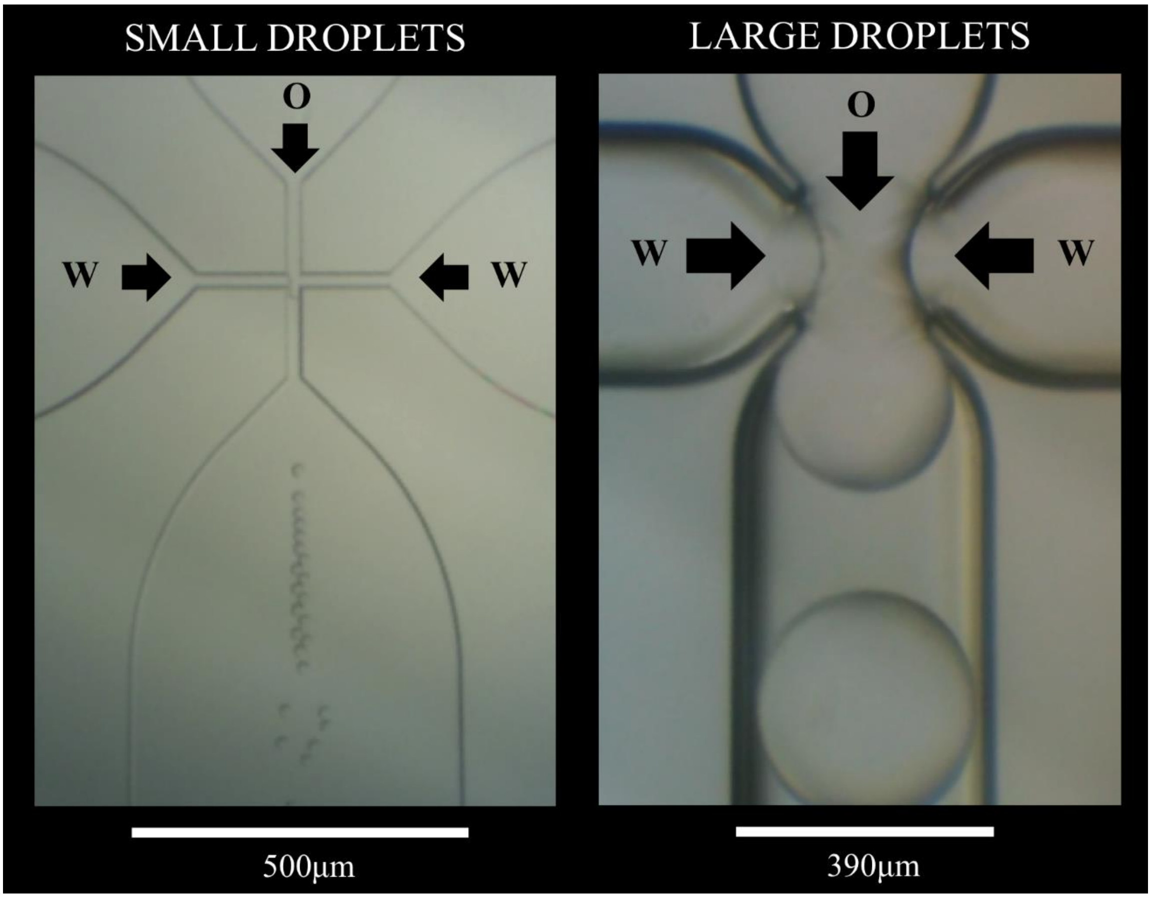

Figure SI2

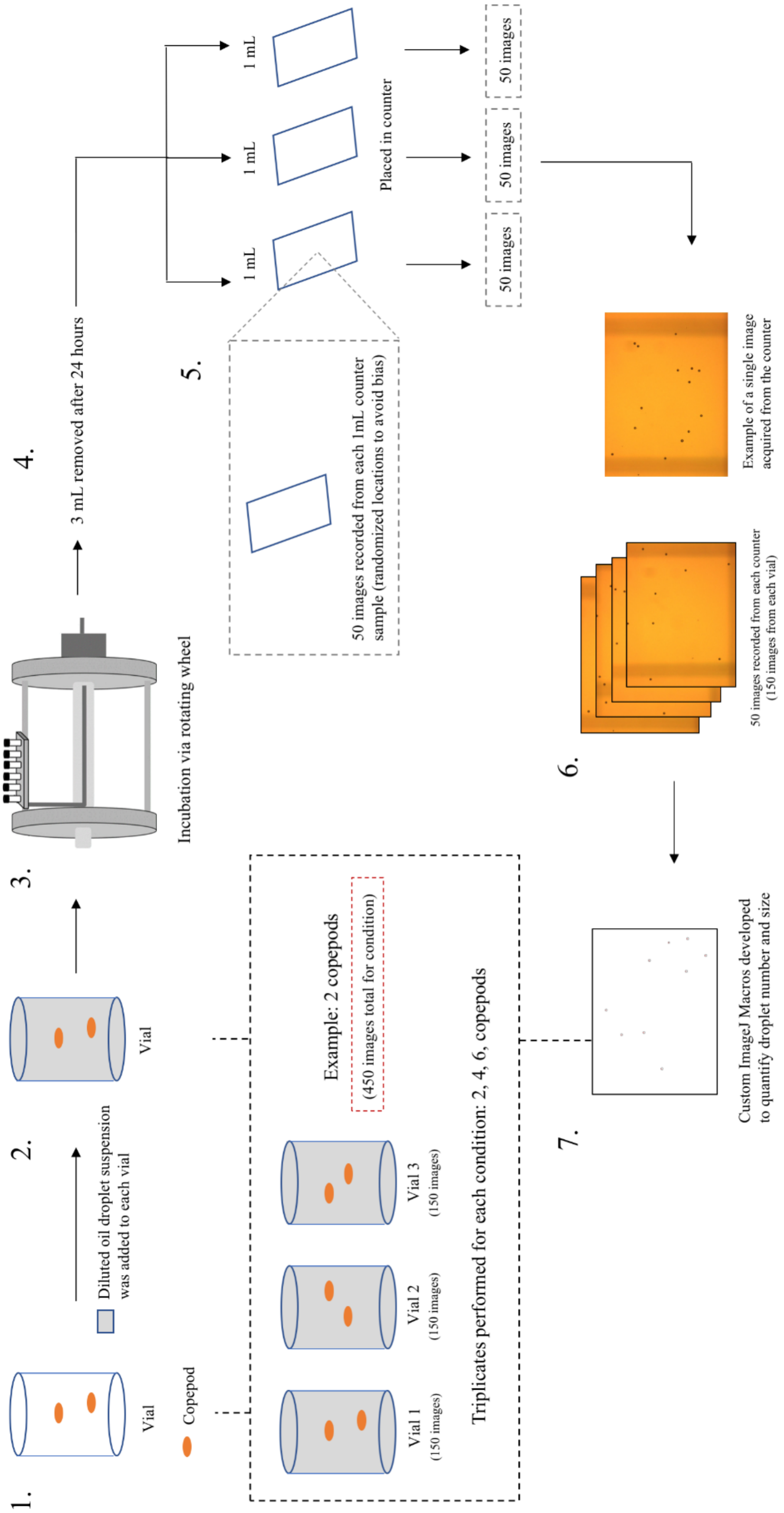

1.
Vial
Copepod
2.
Diluted oil droplet suspension was added to each vial
Vial
3.
Incubation via rotating wheel
4.
3 mL removed after 24 hours
1 mL
1 mL
1 mL
Placed in counter
50 images
50 images
50 images
5.
50 images recorded from each 1mL counter sample (randomized locations to avoid bias)
Example of a single image acquired from the counter
6.
50 images recorded from each counter (150 images from each vial)
Example: 2 copepods
(450 images total for condition)
Vial 1 (150 images)
Vial 2 (150 images)
Vial 3 (150 images)
Triplicates performed for each condition: 2, 4, 6, copepods
7.
Custom ImageJ Macros developed to quantify droplet number and size

Figure SI3

(a)

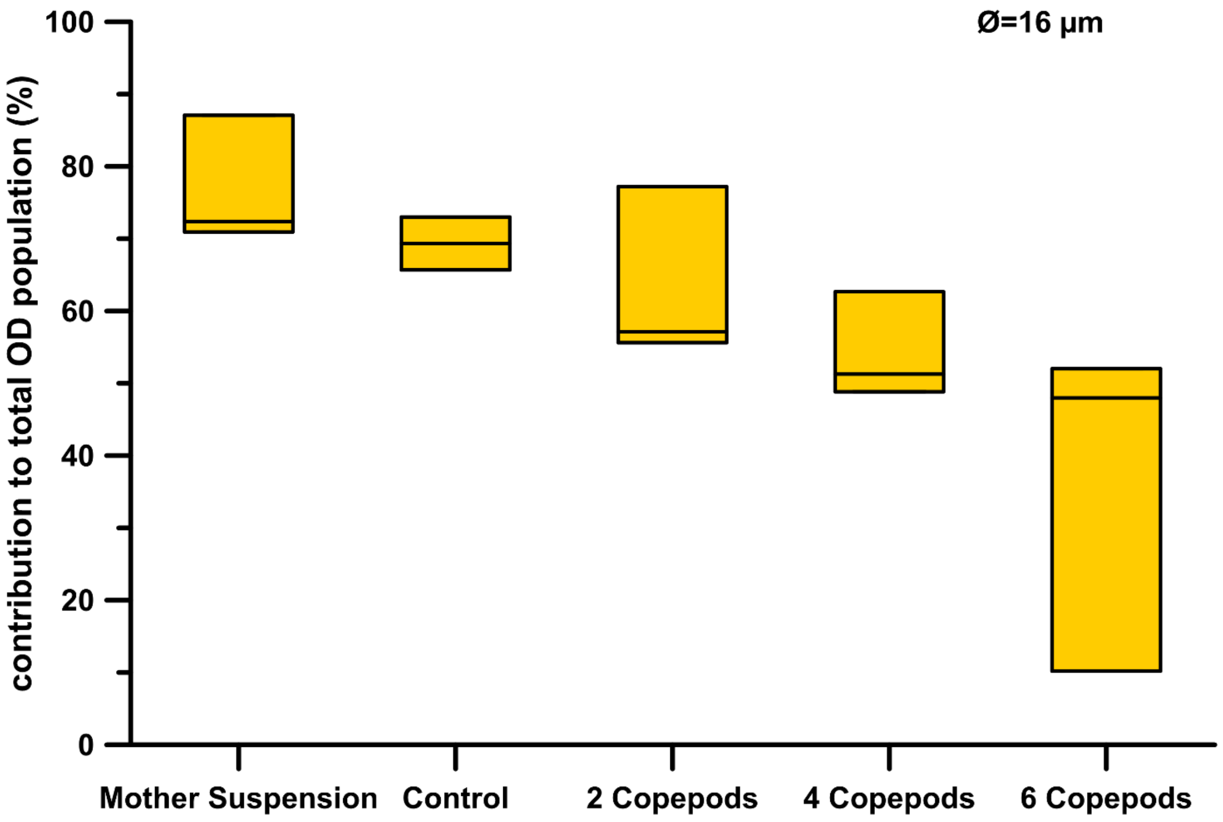

(b)

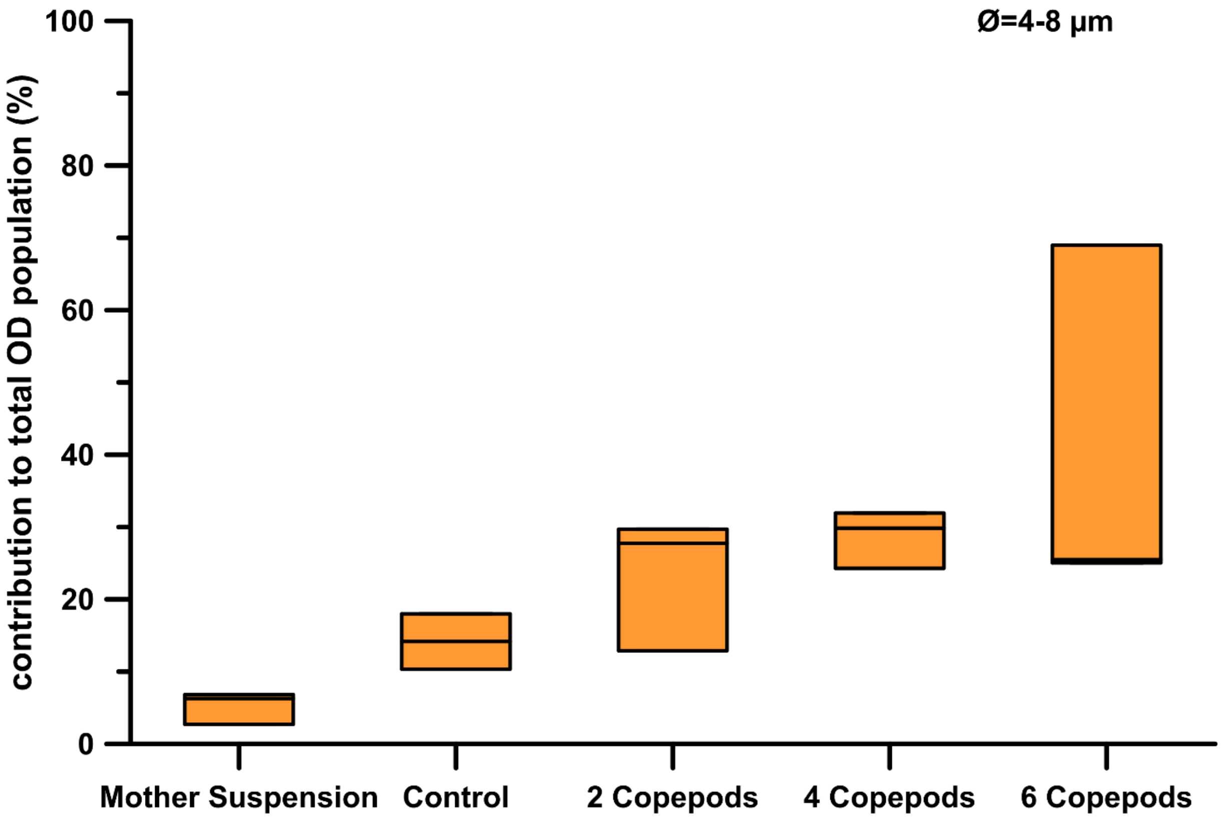

Video Captions

Video SI1: creation of 16 µm Ø oil droplets using the small droplet setup described in the text (see also Table SI1 and Figure SI1).

Video SI2: evidence of Nile red stained oil droplets in the gut of *Paracartia grani* individuals.

Video SI3: evidence of Nile red stained oil droplets in the gut of *Paracartia grani* individuals.
